# Nonlinear Associations of Estimated Pulse Wave Velocity With Hepatic Steatosis and Liver Stiffness in Community-Based People

**DOI:** 10.14740/gr2165

**Published:** 2026-08-26

**Authors:** Tian Hao Wang, Xue Ying Ru, Xiao Xi Zhang, Tian Tian Fu, Hua Yang, Hai Rong Zhu, Zhi Gang Pan

**Affiliations:** a Department of General Practice, Zhongshan Hospital of Fudan University, Shanghai 200032, China; b Department of Geriatrics, The First Affiliated Hospital, Sun Yat-sen University, Guangzhou 510080, China; c School of Global Health, Chinese Center for Tropical Diseases Research, Shanghai Jiao Tong University School of Medicine, Shanghai 200025, China; d Department of Ultrasound, Zhongshan Hospital of Fudan University, Shanghai 200032, China; e These authors contributed equally to this article.

**Keywords:** Metabolic dysfunction–associated fatty liver disease, Artery stiffness, Estimated pulse wave velocity, Hepatic steatosis, Liver elasticity

## Abstract

**Background:**

Metabolic dysfunction–associated fatty liver disease (MAFLD) and its progressive complication, liver fibrosis, have emerged as global public health burdens. Noninvasive tools for integrated assessment of MAFLD and early fibrogenesis remain limited. This study investigated the association between estimated pulse wave velocity (ePWV) and hepatic steatosis/liver fibrosis and assessed ePWV’s utility in evaluating these conditions.

**Methods:**

This cross-sectional study included participants from Shanghai community health centers (March–November 2021) as part of a nationwide MAFLD screening project, “Screening of Metabolic Associated Fatty Liver Disease.” Demographic data, medical history, and laboratory/imaging results were collected. Multiple logistic regression and restricted cubic spline (RCS) curves identified associations between ePWV and MAFLD/liver fibrosis.

**Results:**

A total of 1,116 participants from four community health service centers in Shanghai were enrolled. Among them, 613 had no MAFLD, 232 had light, 202 had moderate and 69 had severe MAFLD, while 886 had no liver fibrosis, 172 had light, 52 had moderate, and six had severe liver fibrosis. Participants with MAFLD or liver fibrosis exhibited higher ePWV values and demonstrated increasing trends across disease severity categories. RCS analyses revealed nonlinear associations between ePWV and both MAFLD and liver fibrosis. When ePWV exceeded 13.26 m/s, the odds ratios (ORs) for MAFLD and liver fibrosis were consistently greater than 1.

**Conclusions:**

ePWV showed nonlinear associations with MAFLD and liver fibrosis. Values above 13.26 m/s were associated with higher odds of both conditions, suggesting that ePWV may serve as a practical vascular marker of hepatic metabolic dysfunction.

## Introduction

Metabolic dysfunction–associated fatty liver disease (MAFLD) is a recently proposed concept that evolved from non-alcoholic fatty liver disease (NAFLD). It describes a chronic, metabolically driven liver condition resulting from overnutrition, insulin resistance, and genetic susceptibility [1]. MAFLD is increasing in prevalence and associated morbidity globally; and China is experiencing particularly high rates [2–4]. Data from over 5.75 million adults who underwent health checkups in China from 2017 to 2022 showed that the prevalence of NAFLD was 44.4% [5]. NAFLD has now replaced viral hepatitis as the leading cause of chronic liver disease in China, and it has become a growing public health concern in the country [3, 6–8].

With respect to liver-related outcomes, the all-cause mortality, and incidence of liver decompensation and hepatocellular carcinoma in patients with MAFLD increased with the progression of hepatic fibrosis [9, 10]. Accordingly, the latest guideline recommended screening and monitoring hepatic fibrosis for patients diagnosed with MAFLD [5]. Although studies have shown that, compared with ultrasonography, the controlled attenuation parameter (CAP) based on transient elastography has higher sensitivity in the diagnosis of fatty liver and that liver stiffness measurement (LSM) based on FibroScan^®^ can be used in evaluation of fibrosis in patients with fatty liver [11, 12], these modalities remain underutilized in primary care settings in China. The diagnosis of fatty liver still relies mainly on ultrasonography and the expertise of sonographers [13].

MAFLD is widely recognized as a systemic metabolic disorder rather than an isolated hepatic condition. Individuals with MAFLD are at increased risk of cardiovascular disease, chronic kidney disease, and other conditions in patients through mechanisms such as lipid accumulation, insulin resistance, and oxidative stress-induced inflammation, compared with the general population [14, 15]. Therefore, current guidelines recommend that patients with MAFLD undergo routine screening for subclinical atherosclerosis [5]. Pulse wave velocity (PWV), especially brachial–ankle PWV (baPWV), is considered a reliable early marker of subclinical atherosclerosis and a “gold standard” indicator of arterial stiffness, with baPWV ≥ 14 m/s commonly used as a diagnostic cutoff [16, 17]. But the measurement of PWV was not generalized in China due to the high expenses and poor comfort [18]. In 2016, European researchers Greve et al developed a formula for estimated pulse wave velocity (ePWV) based on the well-defined correlation of blood pressure and age with PWV [19]. The consistency of ePWV and baPWV was verified in European population and Kailuan study population, and it was found that ePWV also had good predictive value for future cardiovascular events, compared with baPWV [19, 20].

Given the potential value of ePWV as an accessible vascular biomarker, this community-based cross-sectional study examined its associations with hepatic steatosis and liver stiffness and assessed its potential utility in evaluating MAFLD and liver fibrosis.

## Materials and Methods

### Study design

This cross-sectional study was approved by the Ethics Committee of the First Hospital of China Medical University (Approval No.: [2020]330). The study was designed and conducted in accordance with the STROBE (Strengthening the Reporting of Observational studies in Epidemiology) statement, and in compliance with the ethical standards of the responsible institution on human subjects, as well as the Helsinki Declaration [21].

### Study participants

This investigation was part of the national multicenter project “Screening of Metabolic Associated Fatty Liver Disease,” which was carried out in 11 superior hospitals in China such as the First Hospital of China Medical University during 2020 and 2021. Participants in this study were recruited from the general practice clinics of four community health centers in Shanghai by the Department of General Practice, Zhongshan Hospital, Fudan University. Enrollment in Shanghai was carried out from March 2021 to November 2021. For feasibility, we chose the four community health centers that collaborated with Zhongshan Hospital in the standardized training program for general practitioners (GPs), including two centers in urban areas and two in rural areas of Shanghai.

The inclusion criteria were as follows: (1) at least one chronic metabolic disease, such as hypertension, hyperlipidemia and diabetes; (2) age ≥ 18 years; and (3) willing to participate in the study and having signed an informed consent form. The exclusion criteria were as follows: (1) a diagnosed mental illness; (2) severe cognitive or neurological impairments; (3) pregnancy; and (4) any other condition judged by the investigator to render participation unsuitable.

### Data collection and quality control

#### Questionnaire

All participants were required to finish the self-designed High-Risk MAFLD Screening Information Record Form. It comprised four sections: (1) demographic characteristics, anthropometric parameters, and lifestyle factor; (2) medical, current medication and family histories, including cardiovascular diseases, malignancies, MAFLD and other liver disorders; (3) current MAFLD test results; and (4) additional laboratory findings.

#### Physical measurements

Physical check-ups mainly contained the measurement of height, weight, waist circumference (WC), hip circumference (HC), systolic blood pressure (SBP), and diastolic blood pressure (DBP), along with body mass index (BMI) calculation. Height and weight were measured using a corrected weight scales SH-V5 (Zhengzhou Shanghe Electric Technology Co., Ltd.) with participants barefoot and wearing light clothing. BMI was calculated as weight in kilograms divided by height in meters squared (kg/m^2^). WC and HC were measured with a non-elastic tape. Participants stood with feet approximately 25–30 cm apart in a stable position, breathing normally. WC was measured at the natural waist indentation. For HC measurement, the subject stood with feet together and arms relaxed at the sides. The tape was passed horizontally around the pelvis at the level of the symphysis pubis and the greatest gluteal protrusion. Blood pressure was measured in accordance with Chinese guidelines for the management of Hypertension Writing Group of 2018 [22]. Calibrated Omron sphygmomanometers HEM-1000 (OMRON Corporation) were used. After the participant had rested quietly for 5 mins, blood pressure was taken in the seated position, preferably on the left upper arm unless contraindicated.

#### Diagnosis of MAFLD, hepatic steatosis and liver elasticity

According to the latest guidelines, the diagnosis of MAFLD relies primarily on ultrasonography, and excludes excessive alcohol consumption and other secondary causes of hepatic steatosis, with the concurrent presence of at least one component of metabolic syndrome (MS) [5]. Measurements were performed using the FibroTouch FT100 (Wuxi Hisky Medical Technologies Co., Ltd.). Hepatic steatosis was assessed by ultrasonic attenuation parameter (UAP), similar to CAP. UAP values of 244–268 dB/m denote significant steatosis, 269–295 dB/m moderate-to-severe steatosis, and ≥ 296 dB/m severe steatosis [23]. Liver fibrosis is diagnosed using the LSM obtained from FibroScan^®^. An LSM < 8 kPa essentially rules out advanced fibrosis; 8–12 kPa indicates significant fibrosis; > 12 kPa suggests advanced fibrosis; and > 20 kPa is consistent with cirrhosis [24]. All examinations were performed by sonographers with ≥ 3 years of experience, and each result underwent independent secondary review by another qualified sonographer.

#### Laboratory tests

The laboratory tests included alanine aminotransferase (ALT) and aspartate aminotransferase (AST), serum creatinine (SCr), triacylglycerols (TG), total cholesterol (TC), low-density lipoprotein cholesterol (LDL-C), and hypersensitive C-reactive protein (hs-CRP). For the convenience of subjects, all laboratory tests were performed on fasting venous blood collected between 7:00 am and 9:00 am after a minimum 12-h fast (no food or water). The assays of liver function, kidney function and serum lipids were conducted on the Hitachi 3500 automatic analyzer (Hitachi High-Technologies). The routine blood analyses were performed on the Sysmex XML-550 (Sysmex corporation).

#### Quality control

Prior to study initiation, all participating GPs received standardized training and passed competency assessments.

The “High-Risk MAFLD Screening Information Record Form” was completed by trained GPs through direct patient interview and review of existing laboratory results. Before data entry, staff gave each participant a brief orientation covering the study’s purpose, design, confidentiality policy, completion requirements, and key cautions. The form was completed on line; the platform automatically generated a unique participant identification number (ID). Every field must be filled, and any omissions prevented submission. After verifying accuracy, they click “Submit” and the system instantly saved the record. Height, weight, WC and HC were measured by trained nursing staff. Comorbidities, laboratory parameters, and detailed medication use were transcribed from the most recent available laboratory reports or prescriptions issued within the past 6 months. All data were entered twice by two independent operators into an EpiData 3.0 database, followed by automatic consistency checks to ensure accuracy.

### Calculation of ePWV

We adopted the ePWV formulas derived in the Kailuan study population by Ji et al [20].

In general population: ePWV = 9.025 − 0.132 × age + 0.001 × age^2^ + 0.001 × age × MAP − 0.000002 × age^2^ × MAP + 0.029 × MAP

In patients with risks: ePWV = 10.312 − 0.228 × age + 0.003 × age^2^ + 0.002 × age × MAP − 0.000017 × age^2^ × MAP + 0.015 × MAP

MAP denotes mean arterial pressure and was calculated as follows: MAP = diastolic pressure + 0.4 × (systolic pressure − diastolic pressure) [25].

Patients in risks were those with at least one cardiovascular risk factor, including smoking, any MS, history of myocardial infarction, and history of stroke [20]. And MS mainly referred to abdominal obesity (waist ≥ 90 cm in men and ≥ 85 cm in women), hyperglycemia (fasting blood glucose ≥ 6.1 mmol/L or 2-h post-load blood glucose ≥ 7.8 mmol/L, and/or individuals diagnosed with diabetes and undergoing treatment), elevated blood pressure (blood pressure ≥ 130/85 mm Hg and/or individuals confirmed with hypertension and undergoing treatment), and dyslipidemia (fasting triglycerides ≥ 1.70 mmol/L or fasting high-density lipoprotein cholesterol < 1.04 mmol/L) [26]. Patients with none of these risk factors were categorized as general population.

### Sample size

A previous study reported that the PWV in patients with fatty liver was 6.56 ± 1.26 m/s, and that in people without was 5.28 ± 0.87 m/s [27]. Assuming α = 0.05 (two-sided), sample sizes of 38 per group were required. Therefore, the sample size of this study was 86, in case of a 10% loss to follow-up.

### Data analyses

Categorical data was reported as frequencies and percentages (n, %), and Chi-square tests were used to detect significant differences. Continuous data was reported as mean ± standard deviation (SD) and compared using Student’s *t*-tests if on Gaussian distribution; otherwise, medians and interquartile ranges (IQR) were compared using Mann–Whitney U tests.

Variables statistically significant in univariate analyses were included in multivariable models. Before multivariate analysis, collinearity was assessed with variance inflation factor (VIF). Factors with VIF over 10 were deleted. Meanwhile, as age and blood MAP were involved in the calculation of ePWV, we also deleted these three factors. As ePWV was a continuous data and not on Gaussian distribution, we used multinomial logistic regression (MLR) analyses, and calculated odds ratio (OR), 95% confidence interval (CI) and P values. Additionally, the nonlinear correlation between MAFLD/liver fibrosis and ePWV was explored through restricted cubic spline (RCS) curves.

Subgroup analyses were performed among the baseline factors which were statistically significant in MLR. The association between ePWV and MAFLD/liver fibrosis was assessed in each subgroup and illustrated using forest plots.

The dataset contained minimal missing data that did not influence the analysis of the primary outcome, so we did not address the missing values. All statistical analyses were performed with IBM SPSS Statistics, version 27.0 (SPSS Inc) and R 4.3.3. A two-tailed P < 0.05 was considered statistically significant.

## Results

### Participants

**Figure 1:**
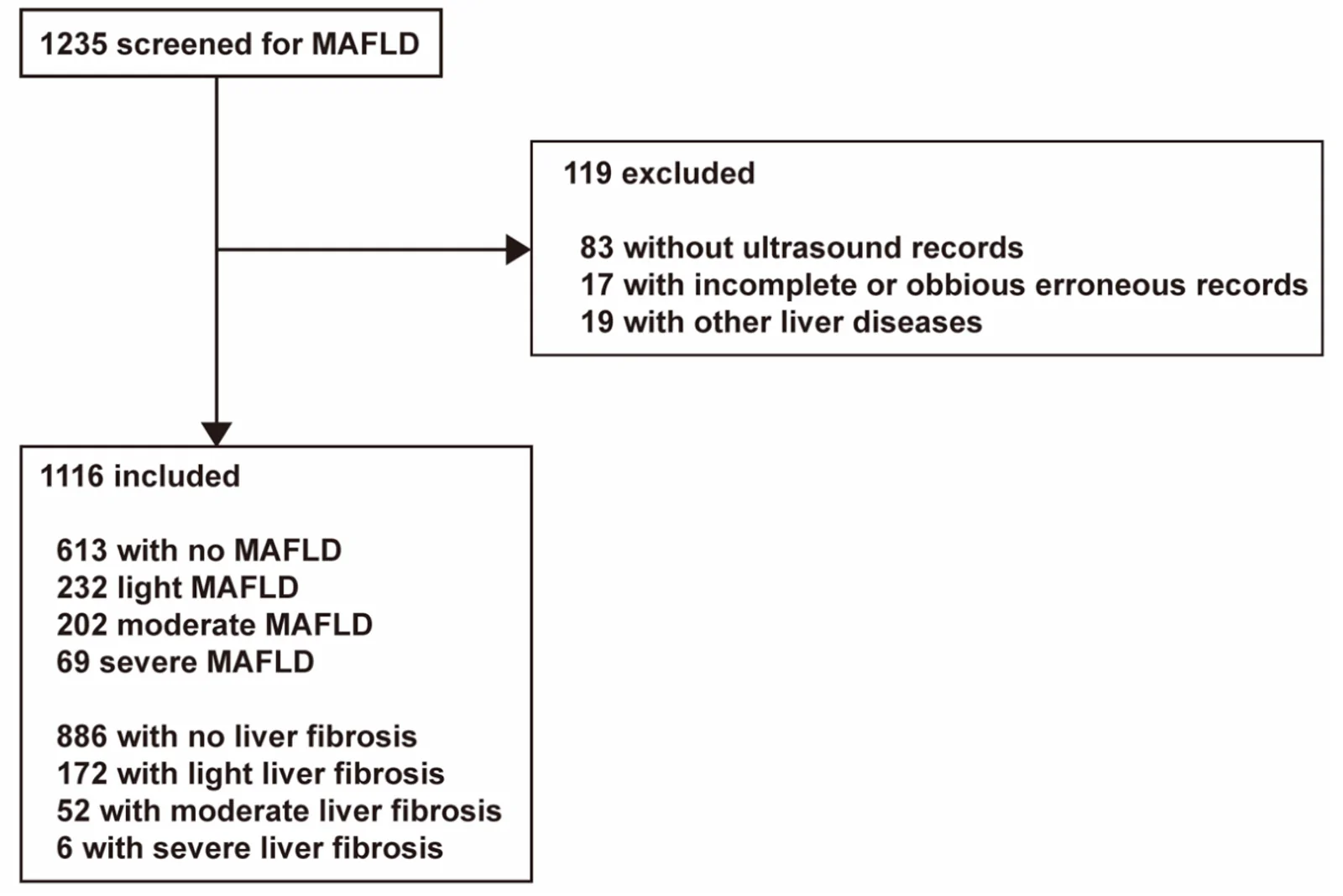
Study flowchart. The flowchart showed that the final analysis comprised 1,116 participants, among whom 613 patients had no MAFLD, 232 had light MAFLD, 202 had moderate MAFLD, 69 had severe MAFLD; 886 had no liver fibrosis, 172 had light liver fibrosis, 52 had moderate liver fibrosis, and six had severe liver fibrosis. MAFLD: metabolic dysfunction–associated fatty liver disease.

Between March and November 2021, 1,235 individuals underwent MAFLD screening in the four selected community health centers. After excluding 88 patients without liver ultrasound reports, 17 with incomplete or obviously erroneous records, and 19 with other liver diseases (viral hepatitis, cirrhosis, hepatocellular carcinoma, etc.), the final analysis comprised 1,116 participants (Fig. 1). Of these participants, 613 did not have MAFLD, whereas 232, 202, and 69 patients had light, moderate, and severe MAFLD, respectively. In addition, 886 patients did not have liver fibrosis, whereas 172, 52, and six patients had light, moderate, and severe liver fibrosis, respectively.

The demographic variables of the 1116 subjects were shown in Table 1. ePWV differed significantly between MAFLD and non-MAFLD groups (Z = −11.616, P < 0.001) and between liver fibrosis and no liver fibrosis groups (Z = −9.293, P < 0.001). Patients with MAFLD were older (*t* = −8.973, P < 0.001), with higher BMI (*t* = −12.496, P < 0.001), WC (*t* = −13.291, P < 0.001), HC (*t* = −8.726, P < 0.001), blood pressure (SBP, *t* = −9.030, P < 0.001, DBP, Z = −6.612, P < 0.001), LSM (Z = −10.899, P < 0.001), TC (*t* =−3.626, P < 0.001), TG (Z = −8.009, P < 0.001), ALT (t = −5.029, P < 0.001), AST (Z = −4.537, P < 0.001) and hs-CRP (Z = -3.410, P < 0.001), and had more males (χ^2^ = 26.762, P < 0.001), current smoker (χ^2^ = 5.669, P = 0.017), dyslipidemia (χ^2^ = 20.909, P < 0.001), hypertension (χ^2^ = 56.541, P < 0.001), chronic obstructive pulmonary disease (COPD) (χ^2^ = 15.416, P < 0.001) and diabetes mellitus (DM)/impaired glucose regulation (IGR) (χ^2^ = 6.289, P = 0.012). Patients with liver fibrosis were older (*t* = -8.165, P < 0.001), with higher BMI(*t* = -7.956, P < 0.001), WC (*t* = -7.690, P < 0.001), HC (*t* = -6.255, P < 0.001), blood pressure (SBP, *t* = −6.630, P < 0.001, DBP, Z = −2.655, P = 0.008), UAP (*t* = -9.871, P < 0.001), TG (Z = −4.244, P < 0.001), ALT (*t* = −3.449, P < 0.001) and AST (Z = −4.029, P < 0.001), and had more males (χ^2^ = 10.267, P < 0.001), dyslipidemia (χ^2^ = 8.963, P = 0.003), hypertension (χ^2^ = 14.769, P < 0.001), coronary heart disease (CHD) (χ^2^ = 9.975, P = 0.002), COPD (χ^2^ = 8.467, P = 0.004) and DM/IGR (χ^2^ = 5.777, P = 0.016).

**Table 1:** Demographic Variables of Subjects

	Non-MAFLD	MAFLD	Z/*t*/χ^2^ (P)	No liver fibrosis	Liver fibrosis	Z/*t*/χ^2^ (P)
Num.n(%)	613 (54.9)	503 (45.1)	—	886 (79.4)	230 (20.6)	—
Light	—	232 (20.8)	—	—	172 (15.4)	—
Moderate	—	202 (18.1)	—	—	52 (4.7)	—
Severe	—	69 (6.2)	—	—	6 (0.5)	—
Age (year)^a^	47 (34, 68)	66.00 (50, 71)	−8.973 (< 0.001)	47 (34, 68)	66 (50, 71)	−8.165 (< 0.001)
Gender^b^			26.762 (< 0.001)			10.267 (< 0.001)
Male	147 (24.0)	193 (38.3)		250 (28.2)	90 (39.1)	
Female	465 (76.0)	311 (61.7)		636 (71.8)	140 (60.9)	
BMI (kg/m^2^)^c^	22.55 ± 3.37	25.03 ± 3.21	−12.496 (< 0.001)	23.25 ± 3.35	25.28 ± 3.74	−7.956 (< 0.001)
WC (cm)^c^	77.15 ± 8.58	83.88 ± 8.23	−13.291 (< 0.001)	79.15 ± 8.82	84.19 ± 8.92	−7.690 (< 0.001)
HC (cm)^c^	90.79 ± 8.00	95.17 ± 8.76	−8.726 (< 0.001)	91.95 ± 8.39	95.87 ± 8.84	−6.255 (< 0.001)
SBP (mm Hg)^c^	120.69 ± 14.30	128.28 ± 13.58	−9.030 (< 0.001)	122.68 ± 14.11	129.65 ± 14.56	−6.630 (< 0.001)
DBP (mm Hg)^a^	75 (70, 80)	78 (72, 82)	−6.612 (< 0.001)	75 (70, 80)	78 (72, 82)	−2.655 (0.008)
HR (bpm)^c^	76.47 ± 8.73	75.91 ± 7.45	1.144 (0.253)	76.15 ± 8.35	76.49 ± 7.53	−0.557 (0.578)
Current drinker^b^	67 (10.9)	62 (12.3)	0.509 (0.475)	98 (11.1)	31 (13.5)	1.044 (0.307)
Current smoker^b^	24 (3.9)	36 (7.1)	5.669 (0.017)	50 (5.6)	10 (4.3)	0.602 (0.438)
Secondhand smoker^b^	152 (25.0)	122 (24.2)	0.084 (0.771)	216 (24.4)	59 (25.7)	0.159 (0.690)
Salt intake (g/d)^a^	6 (6, 6)	6 (6, 7)	−1.848 (0.065)	6 (6, 6)	6 (6, 7)	−0.493 (0.622)
Physical activity (min/d)^a^	60.00 (30.00, 60.00)	60.00 (30.00, 60.00)	−0.323 (0.747)	60 (30, 60)	60 (30, 69)	−0.944 (0.345)
Sedentary time (min/day)^a^	300 (180, 360)	300 (180, 360)	−2.184 (0.029)	5 (3, 6)	5 (3, 6)	−0.615 (0.539)
Medical history^b^						
Dyslipidemia	40 (6.5)	75 (14.9)	20.909 (< 0.001)	79 (8.9)	36 (15.7)	8.963 (0.003)
Hypertension	71 (11.6)	149 (29.6)	56.541 (< 0.001)	154 (17.4)	66 (28.7)	14.769 (< 0.001)
CHD	23 (3.8)	30 (6.0)	2.963 (0.085)	33 (3.7)	20 (8.7)	9.975 (0.002)
COPD	3 (0.5)	19 (3.8)	15.416 (< 0.001)	12 (1.4)	10 (4.3)	8.467 (0.004)
Stroke	10 (1.6)	8 (1.6)	0.003 (0.954)	11 (1.2)	0.7 (3.0)	3.736 (0.053)
DM/IGR	192 (31.3)	194 (38.5)	6.289 (0.012)	291 (32.8)	95 (41.3)	5.777 (0.016)
Hyperuricemia	13 (2.1)	18 (3.6)	2.157 (0.142)	22 (2.5)	9 (3.9)	1.383 (0.240)
UAP (db/m)^c^	210.94 ± 22.73	273.34 ± 20.24	—	233.59 ± 35.02	260.12 ± 40.95	−9.871 (< 0.001)
LSM (kPa)^a^	5.6 (4.8, 6.9)	7.0 (5.6, 8.7)	−10.899 (< 0.001)	5.6 (4.8, 6.9)	7.0 (5.6, 8.7)	—
TC (mmol/L)^c, d^	4.66 ± 0.96	4.90 ± 1.16	−3.626 (< 0.001)	4.76 ± 1.04	4.81 ± 1.16	−0.594 (0.552)
TG (mmol/L)^a, d^	1.1 (0.9, 1.5)	1.4 (1.0, 1.9)	−8.009 (< 0.001)	21 (16, 27)	23 (19, 29)	−4.244 (< 0.001)
LDL-C (mmol/L)^c, d^	2.78 ± 0.88	2.88 ± 0.90	−1.957 (0.051)	2.80 ± 0.87	2.90 ± 0.94	−1.490 (0.137)
HDL-C (mmol/L)^c, d^	1.50 ± 0.51	1.46 ± 0.49	1.521 (0.128)	1.49 ± 0.51	1.46 ± 0.48	0.748 (0.455)
ALT(U/L)^c, e^	21.28 ± 12.27	25.26 ± 14.05	−5.029 (< 0.001)	22.38 ± 12.96	25.75 ± 13.99	−3.449 (< 0.001)
AST(U/L)^a, e^	21 (16, 27)	23 (19, 29)	−4.537 (< 0.001)	21 (16, 27)	23 (19, 29)	−4.029 (< 0.001)
SCr (µmol/L)^c, f^	71.17 ± 42.82	75.26 ± 34.37	−1.725 (0.085)	72.47 ± 41.23	75.10 ± 30.57	−0.904 (0.366)
hs-CRP (mg/L)^a, f^	3.3 (0.8, 5.4)	4.0 (1.4, 6.0)	−3.410 (< 0.001)	3.3 (0.8, 5.4)	4.0 (1.4, 6.0)	−1.732 (0.083)
ePWV (m/s)^a^	12.20 (11.28, 14.68)	14.63 (12.86, 16.24)	−11.616 (< 0.001)	12.20 (11.28, 14.68)	14.63 (12.68, 16.24)	−9.293 (< 0.001)

^a^Continuous data not on Gaussian distribution, reported as median (interquartile range (IQR)) and compared using Mann–Whitney U tests, and the statistic was Z. ^b^Categorical data, reported as n (%), and compared using Chi-square test, and the statistic was χ^2^. ^c^Continuous data on Gaussian distribution, reported as mean ± SD and compared using Student’s *t*-test, with *t* as the test statistic t. P < 0.05 was considered statistically significant. ^d^1,108 subjects were included. ^e^1,106 subjects were included. ^f^1,105 subjects were included. MAFLD: metabolic dysfunction–associated fatty liver disease; BMI: body mass index; WC: waist circumference; HC: hip circumference; SBP: systolic blood pressure; DBP: diastolic pressure; HR: heart rate; CHD: coronary heart disease; COPD: chronic obstructive pulmonary disease; DM: diabetes mellitus; IGR: impaired glucose regulation; LLAs: lipid-lowering agents; UAP: ultrasonic attenuation parameter; LSM: liver stiffness measurement; TC: total cholesterol; TG: triacylglycerols; HDL-C: high-density lipoprotein cholesterol; LDL-C: low-density lipoprotein cholesterol; ALT: alanine aminotransferase; AST: aspartate aminotransferase; SCr: serum creatinine; hs-CRP: hypersensitive C-reactive protein; ePWV: estimated pulse wave velocity.

### Association of ePWV with MAFLD and liver fibrosis

**Figure 2:**
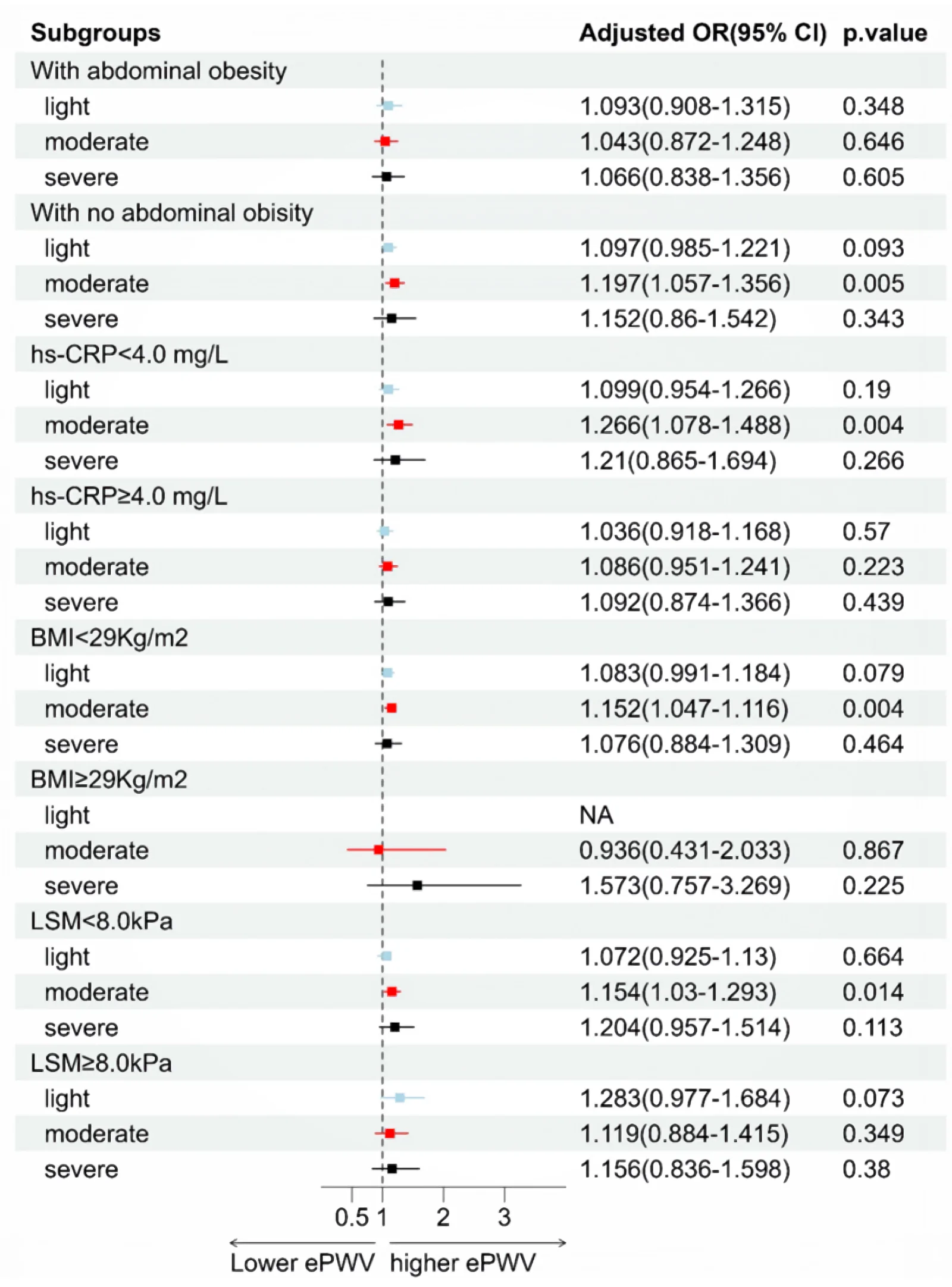
Association of ePWV and MAFLD in different subgroups. The forest plot displays the adjusted odds ratios (ORs) and 95% confidence intervals (CIs) for the association between ePWV and MAFLD severity (light, moderate, and severe) across various clinical subgroups, adjusted for age, BMI, waist circumference, hip circumference, current smoker, sedentary time, dyslipidemia, hypertension, COPD, diabetes, DM/IGR, LSM, TC, TG, ALT, AST and hs-CRP. The vertical dashed line represents the unity line (OR = 1.0). Colored squares indicate point estimates: blue for light, red for moderate, and black for severe MAFLD. Horizontal lines represent the 95% CIs. P < 0.05 is considered statistically significant. Statistical significance (P < 0.05) is achieved when the 95% CI does not cross the unity line. Moderate MAFLD (red) demonstrated significant associations with higher ePWV in several strata (P < 0.05), while light (blue) and severe (black) groups generally showed similar positive trends without reaching statistical significance. MAFLD: metabolic dysfunction–associated fatty liver disease; ePWV: estimated pulse wave velocity; OR: odds ratio; CI: confidence interval; BMI: body mass index; LSM: liver stiffness measurement; COPD: chronic obstructive pulmonary disease; DM: diabetes mellitus; IGR: impaired glucose regulation; TC: total cholesterol; TG: triacylglycerols; ALT: alanine aminotransferase; AST: aspartate aminotransferase; hs-CRP: hypersensitive C-reactive protein (4.0 mg/L was the mean value of hs-CRP in all subjects).

**Figure 3:**
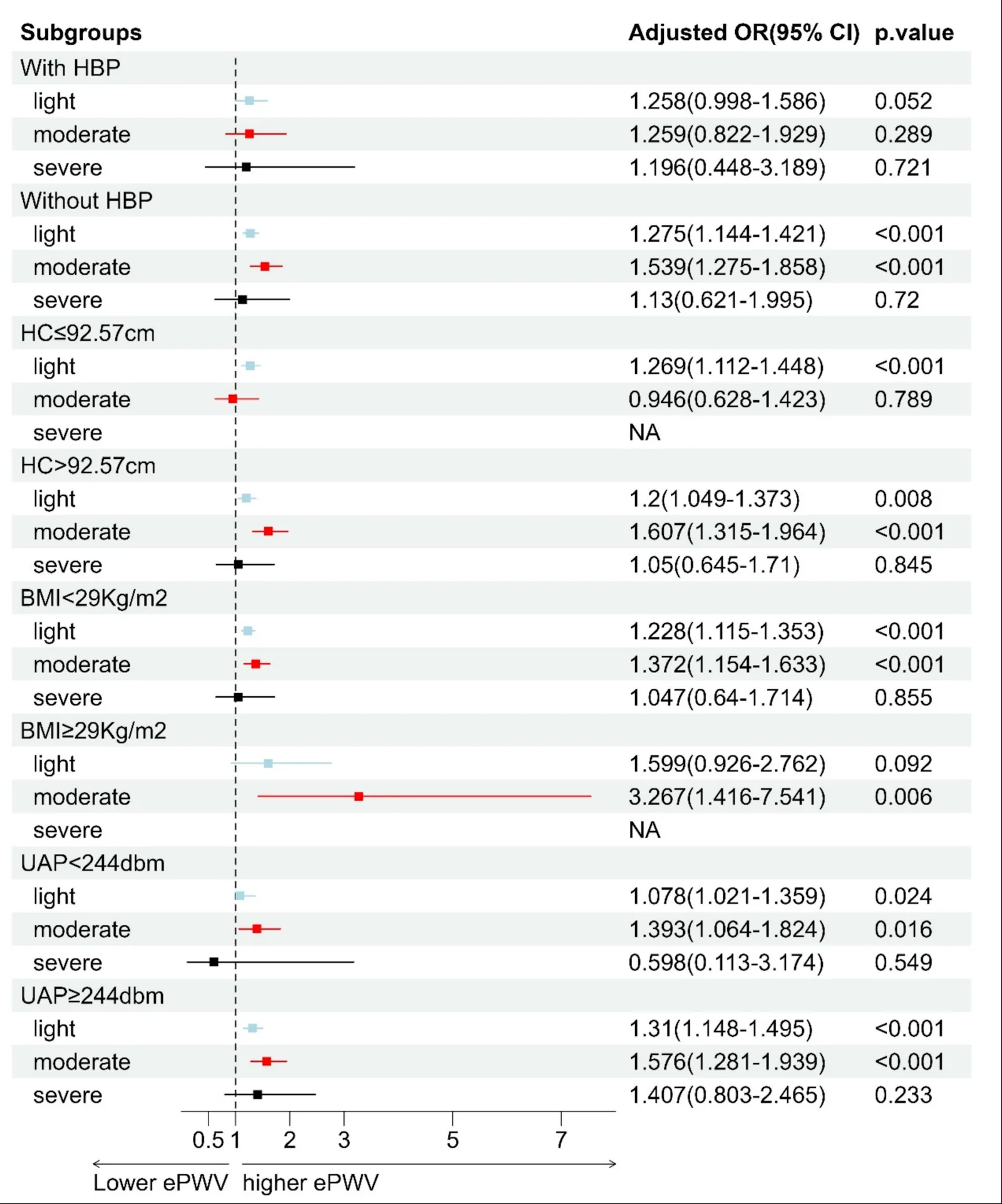
Association of ePWV and liver fibrosis in different subgroups. The forest plot displays the adjusted odds ratios (ORs) and 95% confidence intervals (CIs) for the association between ePWV and liver fibrosis severity (light, moderate, and severe) across various clinical subgroups, adjusted for age, BMI, waist circumference, hip circumference, dyslipidemia, hypertension, CHD, COPD, DM/IGR, UAP, TG, ALT and AST. The vertical dashed line represents the unity line (OR = 1.0). Colored squares indicate point estimates: blue for light, red for moderate, and black for severe liver fibrosis, respectively. Horizontal lines represent the 95% CIs. P < 0.05 is considered statistically significant. Statistical significance (P < 0.05) is achieved when the 95% CI does not cross the unity line. Moderate liver fibrosis (red) and light fibrosis (blue) frequently demonstrated significant associations with higher ePWV (P < 0.05), whereas severe fibrosis (black) showed consistent positive trends but lacked statistical significance. MAFLD: metabolic dysfunction–associated fatty liver disease; ePWV: estimated pulse wave velocity; BMI: body mass index; UAP: ultrasonic attenuation parameter; HBP: high blood pressure; OR: odds ratio; CI: confidence interval; hs-CRP: hypersensitive C-reactive protein; HC: hip circumference (92.57 cm was the median value of HC in all subjects).

Logistic regression analyses demonstrated that participants with MAFLD or liver fibrosis exhibited higher ePWV values, with a stepwise increase across severity categories. Those with moderate level of MAFLD had 1.130 times the ePWV of those without MAFLD (OR = 1.130 (95% CI, 1.025–1.246), P = 0.014). Similarly, those with a moderate level of liver fibrosis had 1.494 times the ePWV of those without liver fibrosis (OR = 1.494 (95% CI, 1.248–1.789), P < 0.001), as shown in Table 2. Subgroup analyses yielded consistent findings (Figs. 2, 3), with most OR point estimates (colored squares) for MAFLD and liver fibrosis positioned to the right of the unity line (OR = 1). Notably, moderate (red) and light (blue) groups frequently demonstrated significant associations with higher ePWV (P < 0.05), while severe (black) group showed similar positive trends without reaching statistical significance.

**Table 2:** Association of ePWV With MAFLD and Liver Fibrosis

	Crude OR (95% CI)	P	Adjusted OR (95% CI)^a^	P
MAFLD (vs. non-MAFLD)				
Light	1.213 (1.134–1.298)	< 0.001	1.047 (0.957–1.145)	0.320
Moderate	1.396 (1.299–1.500)	< 0.001	1.130 (1.025–1.246)	0.014
Severe	1.511 (1.356–1.684)	< 0.001	1.170 (0.992–1.380)	0.062
Liver fibrosis (vs. no liver fibrosis)				
Light	1.294 (1.207–1.388)	< 0.001	1.245 (1.117–1.388)	< 0.001
Moderate	1.427 (1.267–1.606)	< 0.001	1.494 (1.248–1.789)	< 0.001
Severe	1.374 (0.989–1.909)	0.058	1.127 (0.682–1.863)	0.641

^a^In analyses for MAFLD, adjusted for age, BMI, waist circumference, hip circumference, current smoker, sedentary time, dyslipidemia, hypertension, COPD, diabetes, DM/IGR, LSM, TC, TG, ALT, AST and hs-CRP. In analyses for liver fibrosis, adjusted for gender, age, BMI, waist circumference, hip circumference, SBP, DBP, dyslipidemia, hypertension, CHD, COPD, DM/IGR, UAP, TG, ALT and AST. P < 0.05 was considered statistically significant. Reference groups: non-MAFLD for the MAFLD analysis and no liver fibrosis for the liver fibrosis analysis. ePWV: estimated pulse wave velocity; MAFLD: metabolic dysfunction–associated fatty liver disease; OR: odds ratio; CI: confidence interval.

**Figure 4:**
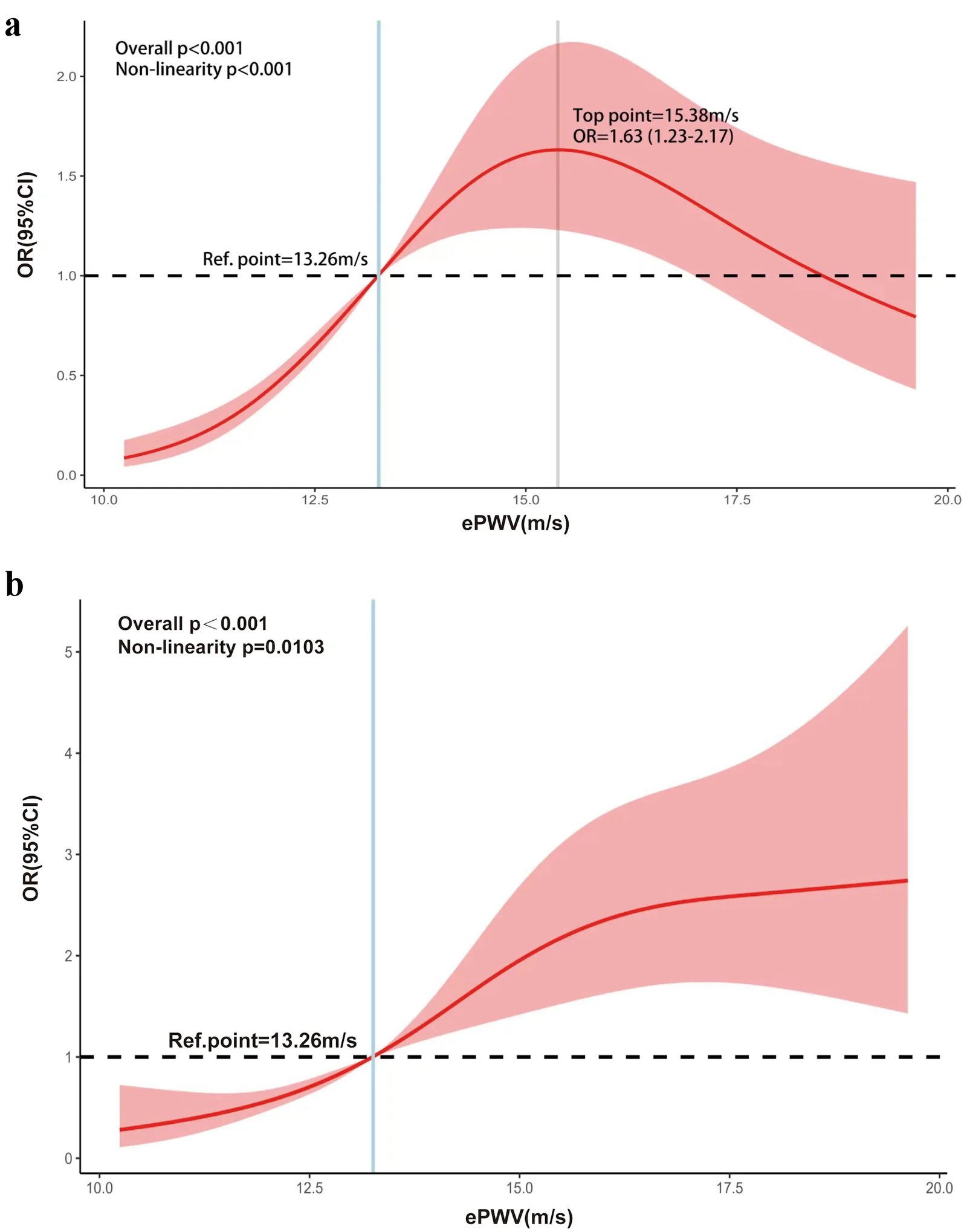
Restricted cubic spline curves for the association between ePWV and MAFLD (a) or liver fibrosis (b). P < 0.05 was considered statistically significant. (a) The figure presented with a reversed “U” shape curve (overall P < 0.001; nonlinearity P < 0.001) and the maximum of OR was showed when ePWV was 15.38 m/s (OR = 1.63; 95% CI, 1.23–2.17). (b) The slope gradually decreased as ePWV increased (overall P < 0.001; nonlinearity P = 0.013). ePWV: estimated pulse wave velocity; MAFLD: metabolic dysfunction–associated fatty liver disease; OR: odds ratio; CI: confidence interval.

The RCS analyses confirmed nonlinear correlations between ePWV and MAFLD/liver fibrosis, as illustrated in Figure 4a, b. In Figure 4a, it was a reversed “U” shape curve (overall P < 0.001; nonlinearity P < 0.001). The maximum of OR was showed when ePWV was 15.38 m/s (OR = 1.63; 95% CI, 1.23–2.17). In Figure 4b, the slope gradually decreased as ePWV increased (overall P < 0.001; nonlinearity P = 0.013). When ePWV exceeded 13.26 m/s, ORs for MAFLD and liver fibrosis were consistently greater than 1.

## Discussion

In this study, we found that ePWV differed significantly among individuals with or without MAFLD and liver fibrosis. ePWV had nonlinearity relationship with MAFLD and liver fibrosis.

Most previous studies showed that individuals with MAFLD exhibit greater arterial stiffness than those without, and that MAFLD was an independent determinant of arteriosclerosis, which aligned with our results [28–31]. For example, in a systematic review and meta-analysis, Giannakodimos et al observed that patients with metabolic dysfunction–associated steatotic liver disease (MASLD) had significantly higher carotid-femoral PWV (cfPWV), baPWV and augmentation index (Alx) than healthy controls (cfPWV: mean difference (MD) = 0.96 m/s; 95% CI, 0.65–1.27 m/s, P < 0.001; baPWV: MD = 78.14cm/s, 95% CI, 60.37–95.90 cm/s, P < 0.001; Alx: MD = 3.85 %, 95% CI, 0.87–6.82%, P = 0.0195) [32].

However, most prior studies considered MAFLD as a binary variable, without examining graded associations across disease severity. Moreover, few studies have examined hepatic steatosis and liver elasticity. One exception was the study by Xin et al, who used noninvasive scores including NAFLD fibrosis score (NFS), Fibrosis-4 index (FIB-4), and aspartate aminotransferase-to-platelet ratio index (APRI), to evaluate hepatic fibrosis and reported that higher baPWV values were associated with a greater likelihood of fibrosis [28]. In our study, patients were stratified into mild, moderate, and severe categories for both MAFLD and liver fibrosis based on UAP and LSM. The study revealed that ePWV increased stepwise with disease severity—specifically, by 13.0% in moderate MAFLD patients compared to those without MAFLD, and by 49.4% in moderate liver fibrosis patients compared to those without liver fibrosis—thereby establishing a quantitative association between disease progression and vascular injury.

Unlike prior studies that assessed atherosclerosis using measured PWV or carotid intima-media thickness (CIMT), we used ePWV, a noninvasive marker derived from routinely available clinical parameters that requires no specialized equipment. RCS analyses identified a threshold of 13.26 m/s, above which the odds of both MAFLD and liver fibrosis were consistently greater than 1. These findings support the potential use of ePWV as a practical risk-stratification marker in primary care settings where conventional PWV measurement is unavailable.

Beyond epidemiological associations, mechanistic studies have explored the biological pathways linking MAFLD to arterial stiffness, providing important complementary evidence [33]. Although the whole pathological mechanism was still unclear, some probable shared risk factors were identified, such as obesity. Obesity contributes to dyslipidemia, insulin resistance, and systemic inflammation, impaired endothelial function, collectively driving plaque formation [34–36]. Hemodynamic changes triggered by endothelial injury (e.g., increased hepatic sinusoidal resistance) further exacerbated hepatocellular hypoxia and fibrosis progression, establishing a vicious cycle of liver-vascular injury. Other factors included hypertension and oxidative stress. Hypertension increased arterial pressure, altered vascular structure and function, facilitating arteriosclerosis [37, 38]. Reactive oxygen species injured endothelial cells and enhanced lipid deposition, hastening atherosclerosis [39, 40]. In addition, genetic polymorphisms may play a role. Specific genetic variants may simultaneously influence the pathogenesis of both MAFLD and arteriosclerosis [41]. As demonstrated in the study by Ren et al, genetic polymorphisms were found to be in linkage disequilibrium with loci linked to arterial stiffness, suggesting that genetic factors may serve as a potential bridge connecting these two conditions [41].

Our study had three main strengths. First, we evaluated the associations of ePWV with both hepatic steatosis and liver stiffness and identified a clinically interpretable threshold using RCS analysis. Second, participants were recruited from community settings, and the analyses adjusted for a broad range of demographic, lifestyle, clinical, and laboratory factors. Third, ePWV can be readily calculated, whereas UAP and LSM require specialized equipment and trained personnel that may not be readily available in primary care settings. Current guidelines also retain imaging, including ultrasonography, as an important component of fatty liver assessment [1, 12]. Therefore, ePWV may provide a practical adjunct for identifying individuals who warrant further liver assessment in primary care.

Several limitations should also be acknowledged. First, the cross-sectional design precluded causal inference regarding the associations of elevated ePWV with MAFLD and liver fibrosis. Second, the exclusion of minors and the recruitment of participants with at least one chronic disease may limit the generalizability of our findings to younger and healthy populations. Third, owing to budget constraints, we did not measure baPWV, preventing a direct comparison between ePWV and baPWV in our subjects. Nevertheless, the validity and reliability of the ePWV calculation have been confirmed in previous Chinese populations, supporting its use as a surrogate marker [20]. In recent years, ePWV alone or in combination with other indices has been widely used to predict stroke, kidney failure and cardiovascular diseases [42–44]. Fourth, there might also be potential confounders. Information on lifestyle, medical history, and medication use was obtained through questionnaires, which might introduce recall bias.

### Conclusions

ePWV was nonlinearly associated with MAFLD and liver fibrosis. When the value of ePWV was over 13.26 m/s, the probability of MAFLD and liver fibrosis increased significantly. These findings suggest that ePWV may serve as a practical vascular biomarker for risk stratification and screening for hepatic metabolic dysfunction in community-based settings. In the future, prospective cohort studies are needed to validate its predictive value for MAFLD and liver fibrosis progression.

## Data Availability

The data supporting the findings of this study are available from the corresponding author upon reasonable request.
